# Differential subsampling with cartesian ordering: A high spatial-temporal resolution dixon imaging sequence for assessment of dural arteriovenous fistula

**DOI:** 10.3389/fneur.2022.1020749

**Published:** 2022-10-18

**Authors:** Xi Chen, Liang Ge, Hailinlin Wan, Lei Huang, Yeqing Jiang, Gang Lu, Jing Wang, Xiaolong Zhang

**Affiliations:** Department of Radiology, Huashan Hospital, Fudan University, Shanghai, China

**Keywords:** dural arteriovenous fistulas, differential subsampling with cartesian ordering, time of flight, cerebral venous thrombosis, hemodynamics

## Abstract

**Objective:**

To evaluate the accuracy of differential subsampling with cartesian ordering (DISCO) in comparison to time of flight (TOF) in detecting dural arteriovenous fistulas (DAVF), cerebral venous thrombosis (CVT) and hemodynamics.

**Methods:**

Sixty-two cases (24 female; aged 14–75; mean age, 51.3 years) were included in our study, with 42 positive and 20 negative cases *via* Digital Subtraction Angiography (DSA). Two neuroradiologists independently evaluated the DISCO and TOF. The sensitivity, specificity, and accuracy of the DISCO and TOF-MRA were individually calculated using DSA as the gold standard. Inter-observer reliability was assessed by using a weighted Cohen's kappa (κ) test; *P* < 0.05 was set as the threshold for statistical significance.

**Results:**

Diagnostic sensitivities of DISCO and TOF for DAVF were 92.86 and 64.29%; specificities were 95.0% and 95.0%; while accuracies were 93.55 and 74.19% respectively. For detected CVT, sensitivities of DISCO and TOF were 100 and 92.31%; specificities were 96.55 and 93.10%; with accuracies 97.62 and 92.86% respectively. In hemodynamic analysis, sensitivity of DISCO for reflux was 95.45%; with a specificity of 95.0%; and accuracy 95.24%. The inter-observer kappa values were 0.857 for DISCO (*P* < 0.001).

**Conclusion:**

DISCO showed a high degree of sensitivity and specificity, suggesting its effectiveness in detecting DAVF with or without CVT. Intracranial hemodynamics can be identified using DISCO in DAVF patients, providing accurate evaluation of cerebral blood flow dynamics during the pre-treatment phase.

## Introduction

Intracranial dural arteriovenous fistulas (DAVFs) are characterized by pathological shunts that directly connect one or more arteries to the cranial sinus, meningeal vein or cortical vein through the endocranium (1–3). DAVFs account for approximately 10−15% of all intracranial arteriovenous malformations (1, 2). Most cranial DAVFs commonly occur by dural venous sinuses (1, 3, 4). Many morphologic and hemodynamic risk factors have been identified to predict DAVF progression, including fistula drainage, cerebral venous thrombosis (CVT), and venous sinus, deep vein or cortical vein reflux (1–4). Preoperative diagnosis of DAVF is of great importance for guiding treatment.

DAVFs on routine examination are usually investigated by structural images acquired using computed tomography (CT) and magnetic resonance imaging (MRI) (3, 5). In addition to digital subtraction angiography (DSA), which remains the gold standard for diagnosing DAVFs, CT and MR angiography (CTA and MRA) are the most useful tools for screening patients suspected of having a DAVF, and for classifying and evaluating the treatment response of these lesions (3, 5). Although the majority of cranial MRA scans, such as time-of-flight (TOF) or phase-contrast (PC) MRA, are performed without contrast agent, artifacts related to slow or turbulent flow can limit their application, leaving room for dynamic contrast-enhanced MRA (DCE-MRA) as an alternative diagnostic modality (6). Additionally, the hemodynamic information provided by ultrafast DCE-MRA is unavailable from TOF or PC MRA. DCE-MRA can be obtained using differential subsampling with cartesian ordering (DISCO) sequencing, and has been used for abdominal, breast, and intracranial aneurysm examinations (6–8). Based on three-dimensional spoiled gradient echo sequence and Dixon-based fat-water separation technology (9, 10), pseudo-random variable density k-space segmentation and k-space sharing reconstruction schemes are adopted to achieve ultrafast acquisition of enhanced images at different time points under the premise of ensuring spatial resolution, and thus realizing the reconstruction of dynamic angiographic images (7).

In this study, we applied DISCO-MRA for assessment of DAVF. By comparing DISCO-MRA with TOF-MRA in detecting fistula drainage, CVT and hemodynamic abnormalities, we evaluated the necessity and advantages of DISCO-MRA in clinical application. We specifically hypothesized that DISCO-MRA can be effectively used for evaluation and depiction of DAVF and CVT, and obtained hemodynamics in comparison to routinely performed TOF-MRA.

## Materials and methods

### Patients

This prospective study was approved by the Institutional Review Board of Huashan Hospital Affiliated to Fudan University (IRB No. KY2019-009). Inclusion criteria contained: a. suspected diagnosis of untreated DAVFs; b. residual, relapsed or cured DAVFs. From September 2020 to August 2021, 64 participants were enrolled. Exclusion criteria included absent of conventional angiography and standard contraindications for MRI or DSA, such as contrast agent allergy, MR incompatible pacemaker, renal insufficiency with estimated glomerular filtration rate < 30 ml/min/1.73m^2^, and other diseases confirmed by DSA, such as arteriovenous malformation (AVM). Two patients were excluded due to incomplete data and the diagnosis of arteriovenous malformation. Finally, 62 participants were included in our study, 42 with DAVF and 20 without: 24 females and 38 males; age range: 14–75 years; mean age: 51.3 years (Table 1), with the range of time between DSA and the MRI examination 0–5 days (mean: 2 days). All participants or legal guardian signed informed consent.

**Table 1:** Demographics, locations and characteristics.

	**Overall**
No. (%)	62 (100)
Mean age [SD (year)]	51.3 (16.7)
Sex	
Male (No.) (%)	38 (61.3)
Female (No.) (%)	24 (38.7)
Without fistula or cured	20 (32.3)
Confirmed fistula or relapsed	42 (67.7)
Locations (No.) (%)	42 (100)
Anterior cranial base	2 (4.8)
Cavernous sinus	12 (28.6)
Superior sagittal sinus	6 (14.3)
Straight sinus	1 (2.4)
Lateral sinus	17 (40.5)
Tentorium	3 (7.1)
Other	1 (2.4)
With CVT (No.) (%)	13 (31.0)
Without CVT (No.) (%)	29 (69.0)
With venous reflux (No.) (%)	22 (52.4)
Without venous reflux (No.) (%)	20 (47.6)

SD, standard deviation; yr, year; No., number; %, percentage.

### Image acquisition

**Figure 1:**
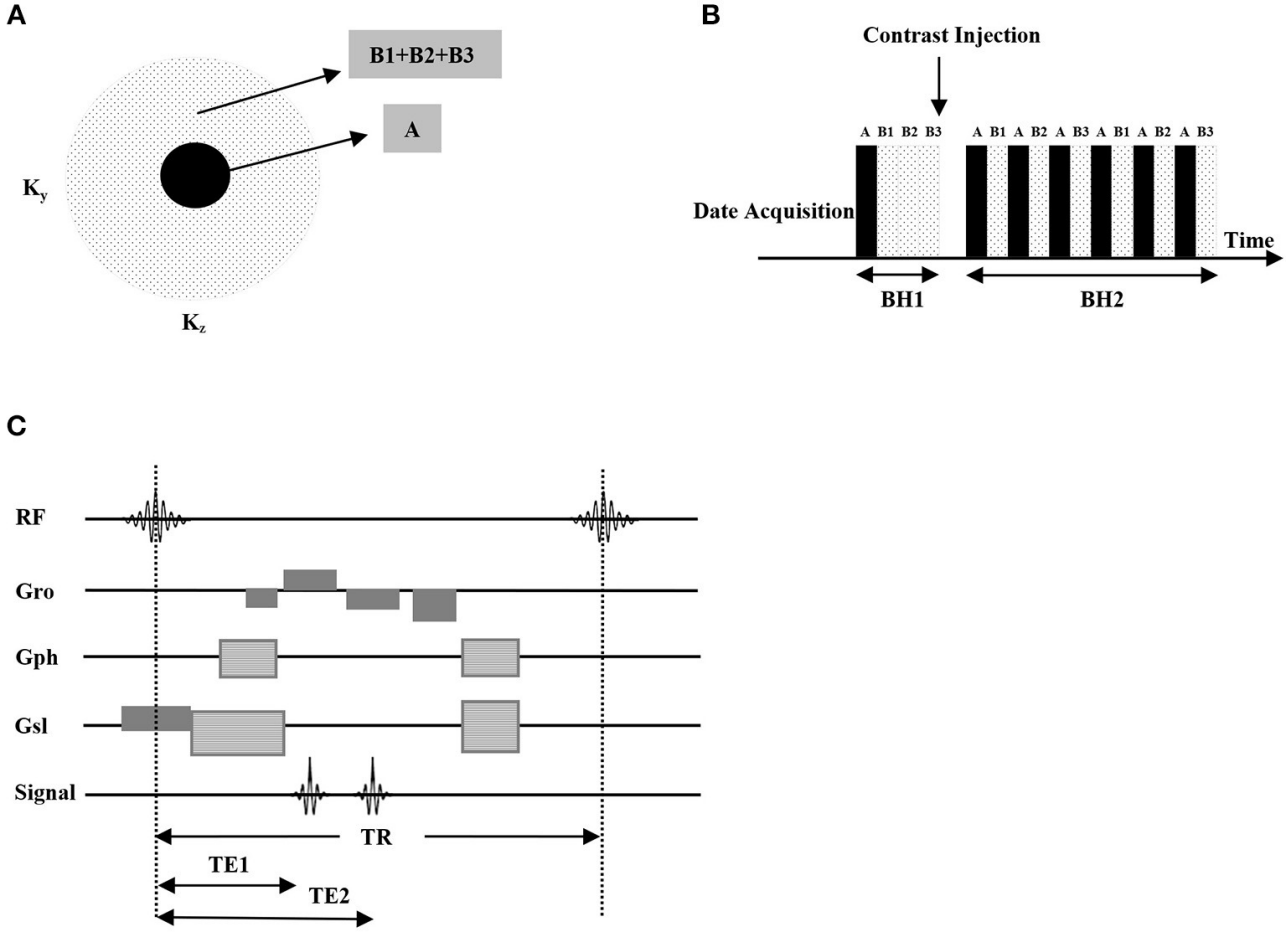
DISCO pseudo-random variable density k-space segmentation **(A)**, the central block region was acquired always and the outer pseudorandom subsampled dotted regions were interleaved (data acquisition schedule is AB1AB2AB3AB1...) **(B)**. The pulse sequence chart of the proposed DISCO sequence, high receiver bandwidths (±83.3 kHz) enabled the acquisition of opposed-phase and in-phase echoes at TE1 (about 1.1 ms) and TE2 (about 2.2 ms) in individual TR, greatly reducing scan times, which is optimal for 3T MRI **(C)**.

All examinations were performed on a 3.0 T MRI scanner (SIGNA Pioneer, GE Healthcare, Milwaukee, WI) equipped with a 32-channel head coil array for signal reception. TOF-MRA sequence was acquired with the following parameters: TR/TE = 25/3.3 ms; flip angle = 25°; FOV = 220 × 220 mm^2^; matrix = 416 × 320; slice thickness = 1.0 mm; 160 slices; bandwidth = 25 kHz; scan duration = 3 min 32 s. TOF-MRA without saturation can show the entire vasculature, both arteries and veins. A DISCO k-space segmentation scheme was utilized with pseudo-random variable density k-space segmentation and a view sharing reconstruction (Figure 1). Specifically, an elliptically ordered central k-space region (16% of total space) was acquired every time, while the peripheral regions were alternatively subsampled with pseudo-random segmentation to minimize the aliasing artifacts. Autocalibrating Reconstruction for Cartesian imaging (ARC) (11, 12) with an acceleration factor of 3 was enabled in both phase-encoding (ky) and slice-encoding (kz) directions. With these settings, a 3D volume covering the entire head with voxel size of 1.1 × 1.4 × 1.4 mm^3^ was obtained. The scanning parameters were: TR/TE = 3.3/1.2 ms; flip angle = 22°; FOV = 280 × 280 mm^2^; matrix = 260 × 200; slice thickness = 1.4 mm; about 136 slices; bandwidth = ±83.3 kHz; temporal resolution = 1.2–2.0 s/phase; scan duration = 2 min−3 min 20 s. To accomplish DCE-MRA, a routine dose (0.05 mmol/kg) of gadodiamide (GE Healthcare Co.) was injected at 4.0 ml/s, flushed immediately by 20 ml saline delivered by a second cylinder.

### Image analysis

TOF-MRA and DISCO-MRA source images were analyzed using Vitrea software (Vital Images, Minneapolis, MI) with three-dimensional reconstruction. Subjective assessment diagnosing DAVF, cerebral venous thrombosis and hemodynamics were performed independently by two certified neuroradiologists, who unaware of the original diagnosis. TOF and DISCO-MRA were read in a random order with the time interval more than 48 h between the two to avoid recall bias. Discrepancies regarding false positives and negatives should be resolved by a consulting doctor.

All data were analyzed in separate sessions and the cases were presented in random order by the study coordinator to avoid recall bias. Fistula drainage was identified by the feeding arteries, the location of fistula and the drainage veins. Diagnosis was assessed by a two-point scoring scale: 0 = negative or unidentifiable; 1 = hint of fistula drainage or identification of fistula drainage. Presence of cerebral venous thrombosis (CVT), defined as dural venous sinus regions with low signal intensity compared to normal intravenous areas, was assessed using source MRA images on another two-point scoring scale: 0 = negative or unidentifiable; 1 = filling defect. DAVF Hemodynamics were also evaluated on a two-point scale: 0 = premature appearance of venous sinus without reflux, or unidentifiable; 1 = reflux of venous sinus, or reflux of cortical or deep vein.

### Statistical analysis

The sensitivity, specificity, accuracy, and diagnostic index (DI) in detection of fistula drainage and CVT were calculated separately for TOF-MRA and DISCO-MRA, using DSA as the gold standard. The ability to detect hemodynamic abnormalities using DISCO-MRA was also calculated. Sensitivity and specificity of the two diagnostic methods were compared by McNemar test. Interobserver reliability was assessed for TOF-MRA and DISCO-MRA utilizing a weighted Cohen's kappa test. All statistical analyses were performed using SPSS 19.0 software (IBM Corp., Armonk, NY). A *P* < 0.05 was considered statistically significant.

## Results

### Diagnosis of dural arteriovenous fistulas

**Figure 2:**
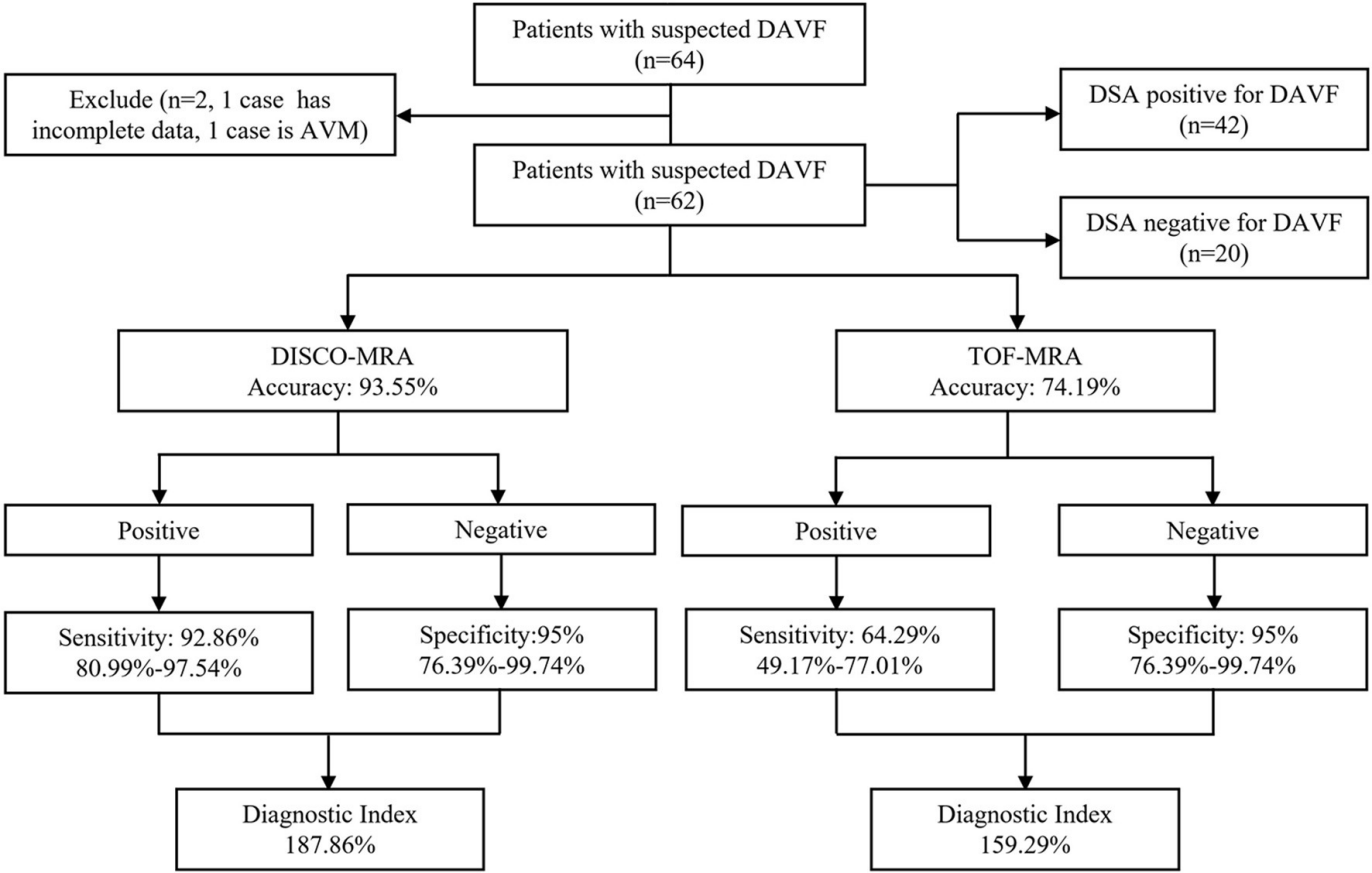
Organizational chart illustrating the patient makeup of this study. The chart includes the accuracy, sensitivity, specificity and diagnostic index (mean with or without 95% confidence interval) of each sequence for detecting DAVF.

**Figure 3:**
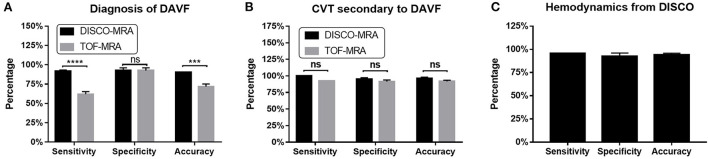
For DAVF detection, the sensitivity of DISCO-MRA (*P* < 0.0001) and accuracy (*P* = 0.0007) were significantly higher than TOF-MRA **(A)**. No significant difference in specificity between DISCO-MRA and TOF-MRA (*P* = 0.1368). With regard to CVT detection, no significant difference in sensitivity, specificity and accuracy between DISCO-MRA and TOF-MRA (all *P* > 0.05) was found **(B)**. For hemodynamics detection, DISCO-MRA had a sensitivity of 95.45% (95% CI: 78.2–99.77%), a specificity of 95.0% (95% CI: 76.39–99.74%) and an accuracy of 95.24% **(C)**. ***P ≤ 0.001; ****P ≤ 0.0001; ns, *P* > 0.001.

**Figure 4:**
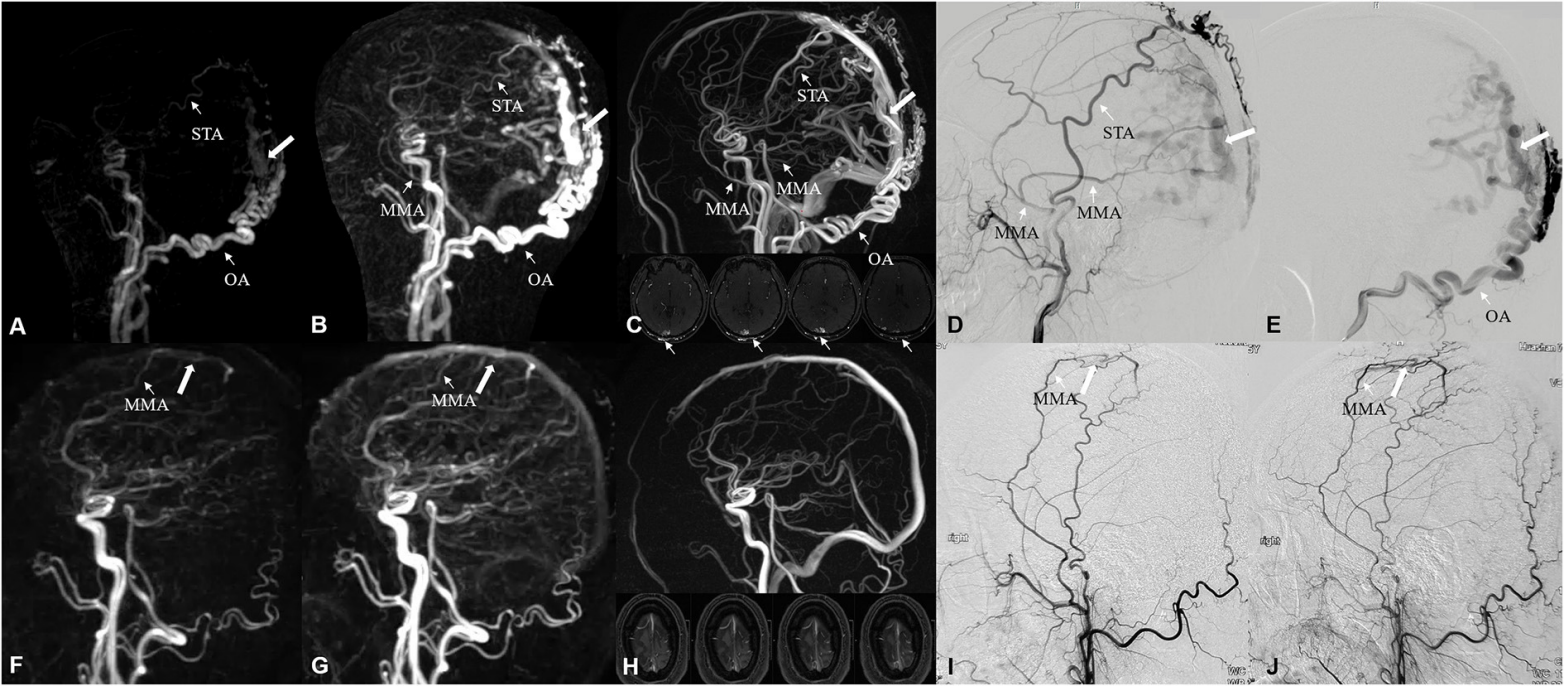
A 48-year-old male suffered from neck and occipital pain for more than 2 years, and aggravated in the past month. Occipital artery (narrow arrow) feeding DAVF, located on the bridge vein (white arrow) of occipital superior sagittal sinus, was identified by DISCO-MRA **(A,B)** and TOF-MRA **(C)**, which was confirmed by DSA **(D,E)**. The feeding arteries were parietal branch of the left STA, posterior convexity branch of the left MMA and transosseous branch of OA. A 45-year-old male suffered from repeated right limb epilepsy accompanied with aphasia for 40 days. Middle meningeal artery (narrow arrow) feeding low-flow DAVF, located on the parietal bridge vein (white arrow), was detected by DISCO-MRA **(F,G)** with TOF-MRA missed diagnosis **(H)**, which was ascertained by DSA **(I,J)**. The feeding artery was parietal branch of MMA. STA, superior temporal artery; MMA, middle meningeal artery; OA, occipital artery.

Among the 62 participants examined, 42 cases were DSA-confirmed DAVF. For DAVF detection (Figures 2, 3A, 4), the sensitivity of DISCO-MRA and TOF-MRA was 92.86% (95% confidence interval, CI: 80.99–97.54%) and 64.29% (95% CI: 49.17–77.01%), respectively. The specificity was 95% (95% CI: 76.39–99.74%) and 95% (95% CI: 76.39–99.74%), while the accuracy was 93.55 and 74.19%, respectively. The false negative rate was 7.14 (95% confidence interval, CI: 2.46–19.01%) and 35.71% (95% CI: 22.99–50.83%), while false positive rate was 5% (95% CI: 0.26–23.61%) and 5% (95% CI: 0.26–23.61%), respectively. The Diagnostic Index (DI) values of DISCO-MRA and TOF-MRA were 187.86% and 159.29%, respectively. The sensitivity (*P* < 0.0001) and accuracy (*P* = 0.0007) of DISCO-MRA were significantly higher than TOF-MRA. No significant difference in specificity between DISCO-MRA and TOF-MRA (*P* = 0.1368) was found (Figure 3A). The interobserver kappa values of DISCO-MRA and TOF-MRA were 0.789 (*P* < 0.001) and 0.771 (*P* < 0.001), respectively, indicating that the results between two observers were highly consistent.

### Diagnosis of cerebral venous thrombosis

**Figure 5:**
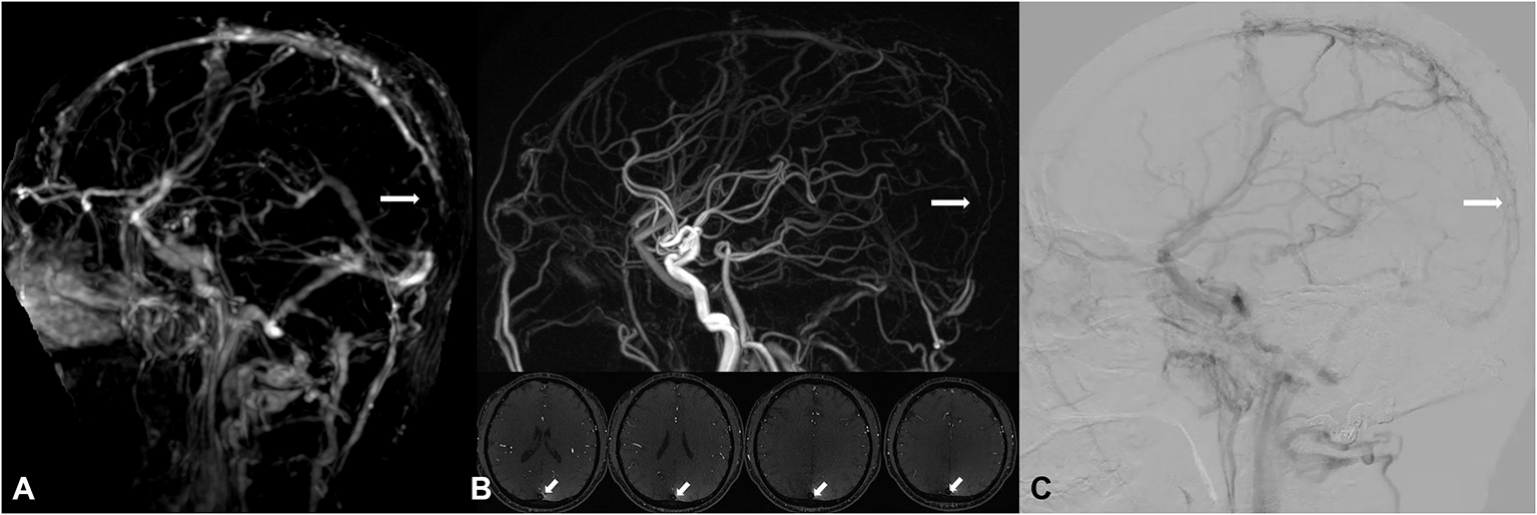
A 51-year-old male, suffered from headache, received interventional therapy for DAVF 5 months ago. Residual DAVF in the superior sagittal sinus was diagnosed, with attendant thrombosis (white arrow) found by DISCO-MRA [lack of signal in the DISCO-MRA venous phase **(A)**] and TOF-MRA [hypointensity in the TOF-MRA slice and reconstructed image **(B)**], and confirmed by DSA [filling defect in DSA venous phase **(C)**].

Among the 42 DAVF positive cases, 13 cases with thrombus and 29 without were found by DSA. With regard to CVT detection (Figures 3B, 5), the sensitivity of DISCO-MRA and TOF-MRA were 100% (95% CI: 77.19–100%) and 92.31% (95% CI: 66.69–99.61%), while the specificity was 96.55% (95% CI: 82.82–99.82%) and 93.10% (95% CI: 78.04–98.77%), with an accuracy of 97.62 and 92.86%, all respectively. The DI values of DISCO-MRA and TOF-MRA were 196.55 and 185.41%, respectively. No significant difference in sensitivity, specificity and accuracy between DISCO-MRA and TOF-MRA (all *P* > 0.05) was found (Figure 3B). The interobserver kappa values of DISCO-MRA and TOF-MRA were 1 (*P* < 0.001) and 0.685 (*P* < 0.001), respectively, indicating that the results between two observers were highly consistent for DISCO-MRA, and moderately consistent for TOF-MRA.

### Diagnosis of hemodynamic abnormalities

**Figure 6:**
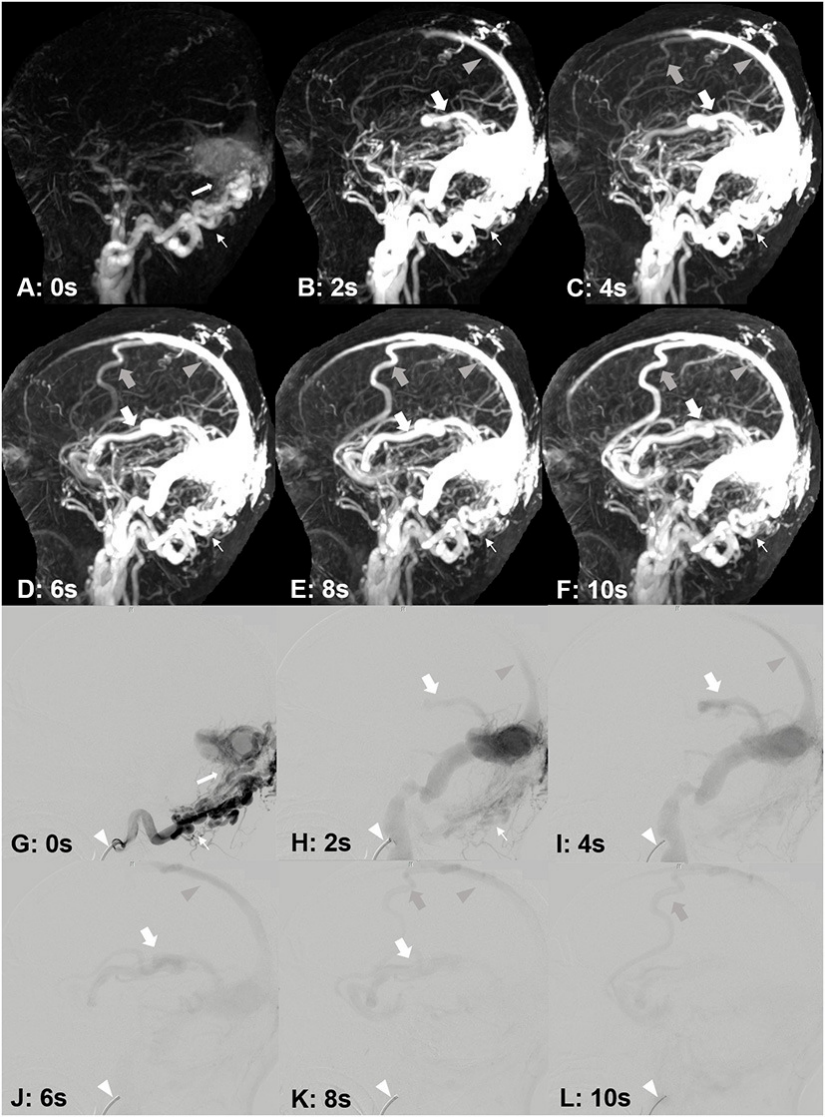
A 33-year-old male, suffered from right tinnitus for 1 year and left headache, dizziness and memory decrease for 1 month. DAVF was identified by DISCO-MRA **(A–F)**. The occipital artery feeding [**(A–F)**, narrow arrows] DAVF occurred in the lateral sinus [**(A)**, white arrow], and the reflux was found in the superior sagittal sinus [**(B–F)**, gray arrow heads], cortical vein [**(C–F)**, gray bold arrows) and Labbe's vein [**(B–F)**, bold arrows]. The interval time of each frame is 2 s. DAVF was confirmed by DSA **(G–L)**. The catheter [**(G–L)**, white arrow head] was placed in right occipital artery [**(G,H)**, narrow arrows]. The fistula occurred in the lateral sinus [**(G)**, white arrow], and the reflux was found in the superior sagittal sinus [**(H–K)**, gray arrow heads], cortical vein [**(K,L)**, gray bold arrows] and Labbe's vein [**(H–K)**, bold arrows]. The interval time of each frame is 2 s.

Among the 42 DAVF positive cases, 22 cases with reflux and 20 without were detected by DSA. For venous reflux detection (Figures 3C, 6), DISCO-MRA had a sensitivity of 95.45% (95% CI: 78.2–99.77%), a specificity of 95.0% (95% CI: 76.39–99.74%), while the accuracy was 95.24% (Figure 3C). The DI value was 190.45%; while the interobserver kappa value of DISCO-MRA was 0.857 (*P* < 0.001), indicating that the results between two observers were highly consistent for DISCO-MRA. Due to the lack of hemodynamic information, TOF-MRA cannot distinguish the direction of blood flow.

## Discussion

Dural arteriovenous fistulas (DAVFs) are generally classified based on the direction of venous drainage (13, 14). Because of its high spatial-temporal resolution, DSA is the gold standard to detect the direction of venous drainage (3, 5). Superselective angiography *via* a catheter is invasive, and can only be performed on a single or dual vessel, which is perfect to determine the hemodynamics of regular DAVF (3, 5). However, in extreme high flow DAVF with abundant feeders, overall hemodynamic pattern and angioarchitecture cannot be observed *via* single feeding arterial angiography, which caused by insufficient venous imaging because of contrast medium dilution. Alternative inspection methods should be considered. Advances in MRI techniques allow for better non-invasive characterization of DAVF in ways that previously were only possible with DSA (3, 5, 6). TOF-MRA without saturation can show the complete vasculature, lacking of hemodynamic information in TOF-MRA can give big impact on the diagnosis of DAVF (3). DISCO-MRA can make up the deficiency through premature venous appearance (6, 7). In this study, we used DISCO sequencing to obtain a high spatial resolution MRA over a large field (whole head) of view with a temporal resolution of 1.2–2 s. The prominent advantage of DISCO over commonly used undersampling view-sharing techniques (such as time-resolved imaging of contrast kinetics: TRICKS also in GE) (15, 16) is due to the adoption of a variable density pseudo-random sampling scheme, which can maximize the temporal resolution and minimize potential artifacts such as signal discontinuities and ghosting commonly seen in alternative organized subsampling techniques (7). Similarly, time-resolved angiography with interleaved stochastic trajectories (TWIST) in Siemens and time-resolved angiography using keyhole (TRAK) in Philips typically use radial sampling schemes, acquiring 3D k-space in round or oval cylinders for temporal resolution scan (17–20).

Our study found that the detection accuracy of DAVF and CVT of DISCO-MRA was significantly higher than that of TOF-MRA. Hemodynamics can be identified by DISCO-MRA, but not by TOF-MRA. For DAVF detection, the DI value of DISCO-MRA is above 170%, and therefore clinically effective in DAVF diagnosis. In contrast, the DI value of TOF-MRA is below the 170% threshold, which is of limited use in diagnosis. The interobserver kappa values were 0.789 for DISCO-MRA and 0.771 for TOF-MRA, indicating that the results between two observers were highly consistent for DISCO-MRA and TOF-MRA. Discrepancies regarding false positives and negatives should be resolved by a consulting doctor, in order to achieve diagnostic consistency. The DI values of thrombus detection were 196.55% for DISCO-MRA and 185.41% for TOF-MRA. Both can be used to identify thrombosis in DAVF cases. The interobserver kappa value was 1 for DISCO-MRA and 0.685 for TOF-MRA, underscoring the much greater consistency of DISCO-MRA to detect venous thrombosis related to DAVFs. The DI value for reflux detection was 190.45% for DISCO-MRA, allowing effective identification of hemodynamic abnormalities of DAVFs. The interobserver kappa values were 0.857 for DISCO-MRA, with the results between the two observers highly consistent for DISCO-MRA. In terms of revealing hemodynamic information, the advantages of DISCO-MRA are becoming apparent. In clinical practice, 1.2–2s temporal resolution is markedly limited in assessment of flow patterns within high flow DAVFs. However, the research on DISCO application of DAVF is still in the exploratory stage, and higher temporal resolution is expected in the future research.

A DAVF is a rare clinical cerebrovascular malformation, an abnormal arteriovenous connecting shunt located in the dura mater, common in middle-aged patients (40–60 years) (21). Many factors are related to the occurrence of DAVFs, such as brain trauma, craniotomy, CVT, infection, tumor, etc (3). The mechanism of its formation remains unclear, and it is believed that it is related to the original opening of arteriovenous communication or neovascularization (1, 3, 4). Usually, clinical symptoms are related to the location of the DAVF and the venous drainage pattern (2–5). DAVF can lead to increased venous pressure, and further to venous congestion and reflux (1, 3). When cortical venous reflux occurs, more obvious clinical symptoms such as intracranial hypertension usually appear (13, 14). Therefore, the identification of venous drainage flow is particularly important. Compared with the submillimeter spatial resolution and sub-second temporal resolution of DSA, DISCO-MRA has significant shortcomings. However, compared to other non-invasive inspection methods, such as CTA or TOF/PC-MRA, DISCO-MRA has clear advantages in spatial-temporal resolution (22, 23). TOF-MRA usually shows abnormal flow-related enhancement of affected dural sinuses and associated draining cortical veins, making it difficult to differentiate feeding arteries from drainage veins. The diagnostic sensitivity of TOF-MRA in DAVF is reported to be about 50% (24). CTA usually carries out non-dynamic contrast enhanced acquisition (dynamic contrast enhanced CTA need excessive radiation), in addition to similar defects with TOF, which application is limited due to bone artifacts and radiation damage (22–24). On the other hand, contrast-enhanced MRA provides multiple imaging findings, such as early appearance of veins and asymmetrically increased vascularity *via* increased signal voids, which can increase the diagnostic sensitivity up to 93% (25, 26). DISCO-MRA, as a type of contrast-enhanced MRA, has great spatial and temporal resolution, making the diagnosis of DAVFs easier.

DAVFs combined with CVT suggest that there is a certain correlation between CVT and DAVF, although the causal relationship between CVT and DAVF is still unclear (3, 4). Studies have suggested that venous hypertension caused by venous thrombosis is the main cause of arteriovenous fistula, possibly related to the increase of venous pressure, leading to the opening of an original arteriovenous pathway or the angiogenesis of brain tissue caused by hypoxia (27, 28). Another view is that CVT may be a secondary event caused by DAVFs (29, 30), due to DAVF induced blood flow disorders, including venous congestion, blood stasis, venous turbulence, etc. In MRA examination, distinguishing the causal relationship between DAVF and CVT presents difficulties. The identification of sinus thrombosis is quite helpful for guiding the treatment (1, 4, 31).

Farb et al. (25) have reported using TRICKS sequence in contrast-enhanced MRA to achieve a temporal resolution of 1.8–2.0 s, depending upon head size and coverage requirements, however this approach sacrifices a degree of spatial resolution. Arteriovenous transit time (AVTT), defined as the interval from initial emergence of the intracranial internal carotid artery to the Rolandic vein (6.01 ± 0.50 s as reported), can reflect the time of blood flow from artery to vein (32–34). Temporal resolution needs to be controlled within AVTT to enable the identification of venous reflux. Therefore, the temporal resolution of DISCO-MRA is controlled at 1.2–2 s, which ensures the spatial resolution and the visualization of blood flow direction. The higher the temporal resolution, the clearer the blood circulation pattern (3, 16, 25). Therefore, MRA technique needs to be improved in terms of spatial-temporal resolution.

This study had certain limitations. First and most importantly, DISCO-MRA has no advantage over DSA in spatial-temporal resolution. Second, TOF-MRA and DISCO-MRA require a longer scan duration than conventional MRI, potentially leading to patient movement, which can cause significant artifacts. Third, differentiation from other intracranial vascular malformations were not mentioned in the study. In the further research, the deficiencies should be resolved.

## Conclusion

DISCO-MRA showed good sensitivity, specificity, and accuracy, suggesting that it will be useful for detecting DAVFs and finding CVT. Intracranial hemodynamic abnormalities can be identified using DISCO-MRA in DAVF patients, providing accurate evaluation of cerebral blood flow dynamics during the pre-treatment phase. Additional studies are needed to determine whether optimized DISCO-MRA sequences can be employed during routine DAVF examination.

## Data availability statement

The original contributions presented in the study are included in the article/supplementary material, further inquiries can be directed to the corresponding author/s.

## Ethics statement

The studies involving human participants were reviewed and approved by Institutional Review Board of Huashan Hospital Affiliated to Fudan University. The patients/participants provided their written informed consent to participate in this study.

## Author contributions

XC and LG conceived and designed the entire review and wrote the paper. HW, LH, and XZ reviewed and edited the manuscript. YJ, GL, and JW contributed to the production of figures. All authors read and approved the manuscript.

## Funding

This study has received funding by the National Natural Science Foundation of China (Grant No. 81771242) and the Shanghai Pujiang Program (Grant No. 20PJ1402200).

## Conflict of interest

The authors declare that the research was conducted in the absence of any commercial or financial relationships that could be construed as a potential conflict of interest.

## Publisher's note

All claims expressed in this article are solely those of the authors and do not necessarily represent those of their affiliated organizations, or those of the publisher, the editors and the reviewers. Any product that may be evaluated in this article, or claim that may be made by its manufacturer, is not guaranteed or endorsed by the publisher.
